# New insights into transparent exopolymer particles (TEP) formation from precursor materials at various Na^+^/Ca^2+^ ratios

**DOI:** 10.1038/srep19747

**Published:** 2016-01-21

**Authors:** Shujuan Meng, Yu Liu

**Affiliations:** 1 School of Civil and Environmental Engineering, Nanyang Technological University, 50 Nanyang Avenue, Singapore 639798; 2 Advanced Environmental Biotechnology Centre, Nanyang Environment & Water Research Institute, Nanyang Technological University, 1 Cleantech Loop, Singapore 637141; 3 Singapore Membrane Technology Centre, Nanyang Environment & Water Research Institute, Nanyang Technological University, 1 Cleantech Loop, Singapore 637141

## Abstract

Transparent exopolymer particles (TEP) are planktonic, organic microgels which play significant roles in cycling of carbon and trace elements, aggregation of particles, feeding and accommodating microbes as well as development of biofilms. However, few studies are available on the mechanism of TEP formation in various water environments. Here we investigate the formation of TEP with alginate blocks as precursors at various Na^+^/Ca^2+^ ratios to simulate the situations in different aquatic environments (e.g. freshwater and seawater). We found that the formation of TEP from precursor materials studied was essentially determined by the Ca^2+^ concentration at a fixed sodium concentration, while Na^+^ at high Na^+^/Ca^2+^ ratio out-competed Ca^2+^ for the binding sites on the precursor molecules, leading to a significantly decreased trend of TEP formation. Our results indicate that a more abundant TEP could be expected in freshwater than in seawater, and we also discuss the engineering implications of the findings.

TEP have been described as a class of transparent particulate acidic polysaccharides that can be visualized via staining with alcian blue, typically with a size larger than 0.4 μm^1^,^2^. Recently, alcian blue stainable particles with a size between 0.05 and 0.4 μm had been classified as colloidal TEP^3^,^4^. TEP are ubiquitous and abundant in many aquatic environments including freshwater and seawater. As a highly surface-active material, TEP are very sticky in nature and highly foldable in physical structure. Because of their high abundance and unique properties, TEP are essentially involved in many processes in aquatic systems. For example, as gel-like, free swimming particles, TEP can enhance the aggregation of solid non-sticky particles in water systems^5^ and they also provide surfaces for microbial colonization^5^,^6^,^7^,^8^. It has been shown that about 0.5–25% of all bacteria present in seawater and freshwater were attached onto TEP^5^. This suggests that free swimming TEP as a carrier can transport bacteria from water phase to another solid surface by adhesion, and evidence shows that TEP can play an active role in the development of aquatic biofilms^9^,^10^,^11^. Therefore, the effect of TEP in the development of reverse osmosis (RO) membrane fouling in seawater desalination has attracted more and more concern^3^,^4^,^12^,^13^.

In general, TEP may be derived from organic substances released by organisms in aquatic environments. Two pathways have been proposed to illustrate the formation of TEP from dissolved organic matter (DOM) in aquatic environments, i.e. abiotic and biotic^5^,^14^,^15^,^16^,^17^. In the biotic pathway, microorganism uptakes DOM and produce TEP via mucus/cell coating detachment, particulate material release or TEP can directly form from detritus of the microorganism during their growth or senescence^5^,^14^,^18^,^19^. In the abiotic pathway, TEP form from precursor substances under specific environmental conditions. TEP precursors are organic substances which could form TEP eventually in aquatic environments. These precursors include dissolved fibrillar polysaccharides released from various planktonic organisms as well as intracellular substances released during lysis or breakage of cells^5^,^11^,^14^. By coagulation, gelation or annealing, such fibrillar polymers form submicron gels which further coagulate to form TEP^5^. It has been believed that abiotic pathway is predominant in TEP formation in various aquatic environments^5^,^11^. Of particular, the abiotic TEP formation significantly depends on the types of precursors and environmental conditions^5^.

It has been observed that the formation of TEP can be promoted by divalent cations (e.g. calcium ion) through bridging with neighboring TEP molecular chains^11^. It should be realized that TEP have been detected in many different ecosystems, including freshwater (rivers, lakes even groundwater), wastewater (including brackish water) and seawater^5^,^11^, where TEP concentrations were found to be highly variable, ranging from μg to mg gum xanthan equivalent per liter^11^. Obviously, these water bodies consist of different salts components (e.g. commonly sodium and calcium ions by quantity) which likely play a critical role in TEP development. For example, sodium concentration relative to calcium is much higher in seawater than in freshwater. However, limited information is currently available on the mechanism of TEP formation in various water environments, especially the effect of sodium ion on TEP formation in the presence of calcium ion at different Na^+^/Ca^2+^ ratios. Therefore, this study aimed to offer new insights into the TEP formation from different precursor materials at various concentrations of calcium and sodium ions. For this purpose, previously studied alginate blocks, namely 1,4-linked β-d-mannuronic acid (MM) and α-l-guluronic acid (GG) and heteropolymeric block (MG), were chosen as model precursors in this study^20^. Alginate widely exists in aquatic environments and it has the characteristics similar to TEP, i.e. rich-acidic-polysaccharides and gelling substance. Alginate has been used as standard substance in TEP determination and previous studies had shown that TEP could form from alginate blocks^2^,^21^,^22^. In addition, different alginate blocks have different chemical and physical properties which in turn provide an opportunity to investigate the mechanisms of TEP formation from diverse precursor materials. This study should be very useful for better understanding TEP formation and abundance in natural aquatic environments and their potential implications in various kinds of membrane-based water and wastewater technologies.

## Methods

### Preparation of sample solutions

MG-, MM- and GG-blocks were fractionated from sodium alginate (Wako, Japan) according to the method by Leal *et al.*^23^. As described in previous studies^21^,^22^, the fraction soluble in 0.3 M HCl represents MG-blocks, whereas the fractions soluble and insoluble at pH 2.85 are MM-blocks and GG-blocks respectively. These fractionated alginate blocks were then used as precursors for TEP development under various conditions.

Precursor solutions were prepared by dissolving MG-, MM- and GG-blocks respectively into ultrapure Milli-Q water (resistivity: 18.2 MΩ.cm at 25 °C) with continuous stirring for 2 hours to make up a final concentration of 50 mg/L. CaCl_2_ (Sigma, USA) and NaCl (Wako, Japan) were used to adjust Ca^2+^ and Na^+^ concentrations in the precursor solutions. All precursor solutions were freshly prepared right before experiments. Although pH was not specifically adjusted, it remained approximately at 6.7 ± 0.3 in all the precursor solutions.

### Determination of TEP in solutions of alginate blocks

**Figure 1:**
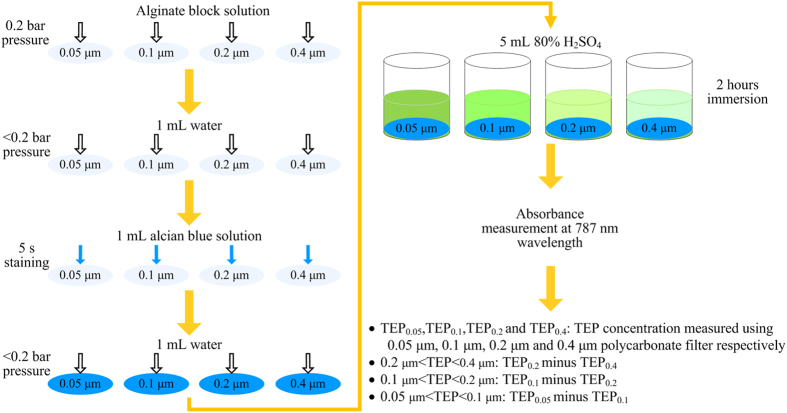
Schematic of the procedure for TEP determination.

In this study, TEP was determined as described in Fig. 1, which is a combination of the methods by Passow and Alldredge^2^ and Villacorte *et al.*^3^,^4^,^24^. This method allows fractionation of TEP to size by means of a series of filtration at different pore sizes of 0.05 to 0.4 μm.

The prepared precursor solutions were first filtered through a series of polycarbonate filters (Whatman, United Kingdom) of pore size 0.05, 0.1, 0.2 and 0.4 μm at a constant vacuum of 0.2 bars, followed by filtration of 1 mL of Milli-Q water for washing out the remaining salinity^24^. Subsequently, the TEP retained on the filters were stained by 1 mL of alcian blue solution at a concentration of 0.02% of alcian blue 8 GX (Sigma, USA) in 0.06% acetic acid (pH 2.5). The alcian blue solution used in this study was filtered right before use with 0.05 μm polycarbonate filter to avoid the dye coagulation^2^,^4^. After 5-s reaction, excess dye was then removed by vacuum filtration at a pressure of about 0.2 bars and was then rinsed by 1 mL of Milli-Q water. Rinsed filters were further immersed in 5 mL of 80% H_2_SO_4_ solution with regular shaking over a period of 2 hours. Alcian blue dissolved in sulfuric acid exhibited a light green color, and the absorbance of the sulfuric acid solution with eluted alcian blue was measured using a UV–Vis spectrophotometer (Shimadzu UV-2501PC, Japan) at 787 nm wavelength in a 1 cm cuvette. Absorbance corrections due to stain adsorption onto filter and interference associated with solution salinity were also performed according to the procedures proposed by Villacorte *et al.*^4^,^24^. Three replicates were conducted for each sample and every sample was measured at least three times. Averaged TEP concentration was reported. Gum xanthan (Sigma, USA) was used as a standard substance of TEP for calibration, thus TEP concentration was ultimately expressed as mg gum xanthan equivalent per liter of solution, i.e. mg X_eq_.L^−1^. Total organic carbon (TOC) measurements were employed to determine the amount of xanthan gum retained by the polycarbonate filters when performed the calibration^3^. It should be noted that the term TEP used in this study includes all alcian blue-stainable particles that can be retained by 0.05 μm polycarbonate filter, unless stated otherwise.

### Microscopic observation of TEP

To visualize the TEP derived from the precursors (e.g. various alginate blocks), a bright-field microscope (Keyence, Japan) was employed. The fresh sample solutions were prepared prior to observation as described above. In order to visualize TEP with the bright-field microscope, precursor solutions were stained by freshly pre-filtered (0.05 μm polycarbonate filter) alcian blue solution as presented above. Stained samples were then observed under the microscope. For each sample, about 20 images were randomly taken.

The micro-structures of TEP derived from MG-, MM- and GG-blocks at different Na^+^/Ca^2+^ ratios were also observed by a field emission scanning electron microscopy (FESEM) (Jeol JSM-7600F, Japan). Although TEP were freeze dried prior to microscopic observation, this microscopic technique could still provide direct visualizations of evidence of TEP micro-structures. 10–50 mL of sample solutions prepared as described above were filtered through 0.1 μm polycarbonate filters (Whatman, United Kingdom) at a constant pressure of 0.2 bars and was then rinsed by 1 mL of Milli-Q water. Filters with retained alginate blocks were completely freeze-dried completely in a freeze dryer (Christ, Germany) for further examination. All samples were observed at least three times and 8–10 images were randomly recorded each time.

### Viscoelasticity of TEP formed at different Na^+^/Ca^2+^ ratios

A XE-100 AFM (Park Systems, Korea) was employed to determine the stickiness and micromechanical characteristics of TEP developed from GG-blocks as precursor at different Na^+^/Ca^2+^ ratios. For force measurements on TEP, TEP films were prepared by filtering 1 mg X_eq_ TEP onto 0.1-μm polycarbonate filters at a constant pressure of 0.2 bars. Subsequently, the filter with retained TEP was carefully glued onto a holder using double-sided sticky tape with TEP film upwards. All TEP samples were prepared right before the force measurements. To prevent TEP film dehydration, the TEP samples were analyzed in a liquid cell filled completely with the test solution of interest. The chemistry composition of the test solution was the same as that used in the corresponding TEP samples, while a clean 0.1-μm polycarbonate filter was used as a reference surface. The clean filter was first soaked in Milli-Q water for 24 hours and 50 mL Milli-Q water was then filtered through it just before the measurement. Force measurements on the clean filters were conducted with all test solutions.

The force measurements were conducted at room temperature of 22 ± 2 °C. Surface architecture was first imaged using non-contact mode to minimize the contact of the tip with the TEP film and subsequently a suitable position on the surface was located for force measurements. For the force measurements, the scan rate was set at 1 Hz with image resolution at 512 data number per trace. The force measurements were approached for an area of 5 × 5 μm^2^ at a respective forward and backward speed of 0.3 μm/s. A minimum of four different places on the TEP film was measured and 16 force measurements were conducted at each location. XEI 1.8.0.Build32 software was used to flatten image as well as to calculate the maximum retract force (adhesion) and total adhesion energy.

## Results

### TEP formation at various Ca^2+^ concentrations

**Figure 2:**
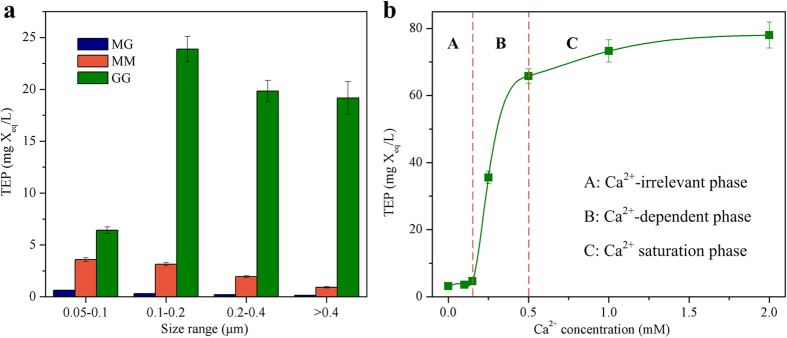
TEP concentrations formed from alginate blocks. (**a**) TEP levels in solutions of MG-, MM- and GG-blocks. Experimental conditions: MG-, MM- and GG-blocks concentration = 50 mg/L, Na^+^ concentration = 10 mM, Ca^2+^ concentration = 1 mM; (**b**) The total TEP concentrations (TEP retained by 0.05 μm polycarbonate filters) formed from GG-blocks with increasing Ca^2+^ concentration. Experimental conditions: GG-blocks concentration = 50 mg/L, Na^+^ concentration = 10 mM. Data are shown as the mean ± s.d., n = 6 independent measurements of the TEP concentrations.

Figure 2a shows the TEP formation with MG-, MM- and GG-blocks as precursor materials at a Ca^2+^ concentration of 1 mM and Na^+^ concentration of 10 mM. For TEP with different sizes ranging from 0.05 μm to 0.4 μm and above, the highest concentration was observed in GG-blocks solution, while the lowest concentration was achieved in MG-blocks solution. For example, the TEP concentrations developed in the solution of GG-blocks are 10-, 75-, 97- and 138-times higher than those in MG-blocks solution in respective TEP size range of 0.05–0.1 μm, 0.1–0.2 μm, 0.2–0.4 μm and >0.4 μm. The total TEP concentrations (TEP retained by 0.05 μm polycarbonate filters) on average in MG-blocks solution was about 1.29 mg X_eq_/L, while 9.59 mg X_eq_/L and 73.25 mg X_eq_/L found in MM-and GG-blocks solutions respectively. At given Na^+^ and Ca^2+^ concentrations, such tendencies of TEP formation from MG-, MM- and GG-blocks is probably due to the differences in the the stiffness of these precursor materials, which indeed have shown to be in the order MG<MM<GG^25^,^26^. The MG-blocks are highly flexible and have a tendency to bind to water molecules instead of forming complexes with divalent cations^25^, such as calcium ion used in this study. As a result, the least TEP was formed in MG-blocks solution. As the rigidity of MM-blocks was higher than that of MG-blocks, MM-blocks preferably react with calcium ion, leading to more TEP produced. GG-blocks, which possess the highest rigidity among three types of alginate blocks, can strongly bind with calcium ion, forming a three dimensional structure through ionic interactions with Ca^2+^, known as “egg-box” structure^20^,^27^. The biaxially linked G residues of linear GG-blocks form rows of cavities which can accommodate calcium ions, and facilitates the development of an egg-box-like network. Thus, the highest TEP level was achieved in the GG-blocks solutions. These suggest that the tendency of TEP formation is greatly determined by the physic-chemical property of precursor materials under defined conditions.

Figure 2b further shows TEP formation from GG-blocks at various Ca^2+^ concentrations with a fixed Na^+^ concentration of 10 mM. According to Ca^2+^ concentration, the phases pertaining to TEP development can clearly be derived from Fig. 2b: (i) at low Ca^2+^ concentration (0 to 0.015 mM), TEP formation was not sensitive to Ca^2+^ concentration; (ii) at medium Ca^2+^ concentration of 0.15 to 0.5 mM, the formation of TEP from GG blocks appeared to be proportionally related to Ca^2+^ concentration, and as the result, a sharp increase in TEP concentration was observed in this phase; (iii) at high Ca^2+^ concentration of 0.5 to 2.0 mM, further increase of the Ca^2+^ concentration had insignificant effect on TEP formation from GG-blocks at a given concentration of GG-blocks. These results indeed provide direct evidence showing the dependence of TEP formation on calcium ion for a given precursor material, i.e. Ca^2+^ should play a critical role in TEP formation.

### TEP formation at various Na^+^ concentrations

**Figure 3:**
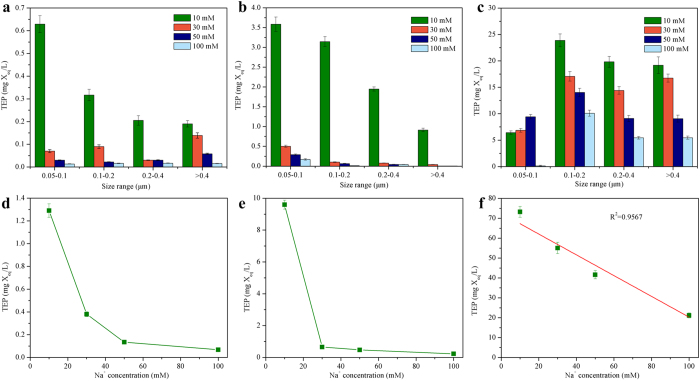
TEP concentrations derived from alginate blocks with increasing Na^+^ concentration. TEP levels in each size range derived from (**a**) MG-blocks, (**b**) MM-blocks and (**c**) GG-blocks at diverse Na^+^ concentrations. The effect of increasing Na^+^ concentration on the total TEP concentration (TEP retained by 0.05 μm polycarbonate filters) derived from (**d**) MG-blocks, (**e**) MM-blocks and (**f**) GG-blocks. Experimental conditions: MG-, MM- and GG-blocks concentration = 50 mg/L, Ca^2+^ concentration = 1 mM, Na^+^ concentrations are 10 mM, 30 mM, 50 mM and 100 mM respectively. Data are shown as the mean ± s.d., n = 6 independent measurements of the TEP concentrations.

Figure 3 shows that increasing Na^+^ concentration at a fixed Ca^2+^ concentration of 1 mM leads to a remarkable reduction in TEP formation from all the three precursor materials, i.e. MM-, MG-, and GG-blocks. Such a trend is completely different from that observed at various Ca^2+^ concentrations with a fixed Na^+^ concentration (Fig. 2b). In the case where MG-blocks served as precursor, the concentration of TEP developed at 10 mM Na^+^ was about 47-, 14-, 12- and 9-times higher than those at a Na^+^ concentration of 100 mM for TEP in the size range of 0.05–0.1 μm, 0.1–0.2 μm, 0.2–0.4 μm and >0.4 μm. The more significant impact of Na^+^ on the formation of TEP with MM-blocks as precursor was observed in Fig. 3b. At a Na^+^ concentration of 100 mM, extremely low-level TEP was developed with both MG- and MM-blocks as precursor. Similar to MG- and MM-blocks, total concentration of TEP developed from GG-blocks tended to decrease with increasing Na^+^ concentration from 10 mM to 100 mM at a given Ca^2+^ concentration of 1 mM (with exception of 0.05–0.1 μm size range). In contrast to Ca^2+^ (Fig. 2b), it appears from Fig. 3 that Na^+^ exerts a significant adverse effect on TEP development from all precursor materials studied.

As discussed above, Ca^2+^ may serve as a bridge to connect precursor molecules in the complex TEP structure. The binding sites at the precursor molecules (alginate blocks) are negatively charged, thus an electrical competition between positively charged Na^+^ and Ca^2+^ exists for the binding sites. Increased Na^+^ concentration can out-compete Ca^2+^, thus prevents the Ca^2+^ bridging and decreases the TEP formation. As shown above, in the cases where MG- and MM-blocks served as precursors, the bindings between calcium ions and these precursors are weaker than that between GG-blocks and calcium ions. Therefore, the bridging effect by calcium ion in MG-and MM-blocks is weakened or even disappeared at elevated Na^+^ concentration, and this in turn explains significantly reduced TEP formation as shown in Fig. 3d–e. Unlike MG- and MM-blocks, GG-blocks can bind with calcium ion more strongly, thus the adverse effect of Na^+^ on TEP formation from GG-blocks is less significant (Fig. 3f).

In general, freshwater (e.g. lake, river and reservoir) often contains less Na^+^ than seawater. As revealed in this study, presumably more TEP could form in fresh water than in seawater at the same level of precursor materials. This is supported by previous studies showing that the highest TEP concentration reported in seawater was about 40% and 3.6% of those in freshwater and wastewater, respectively^11^,^28^,^29^. It should be realized that the occurrence of TEP-associated biofilm development would be more frequent in freshwater and wastewater than in seawater. These may imply that TEP-associated fouling would pose a more serious challenge in wastewater reclamation by membrane than in seawater desalination.

### Microscopic observation of TEP

**Figure 4:**
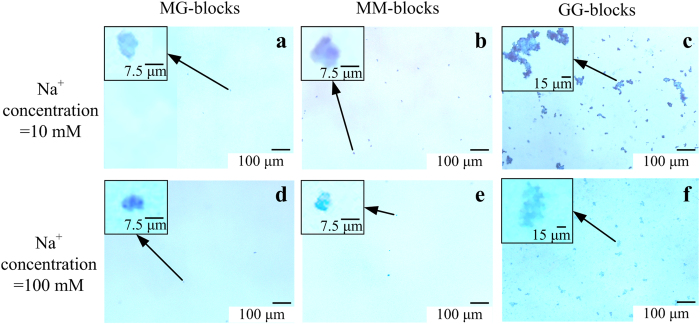
Microscopic observation of the effect of increasing Na^+^ concentration on TEP derived from alginate blocks. TEP formed from MG-, MM- ang GG-blocks at 10 mM (**a**–**c**) and 100 mM (**d**–**f**) Na^+^ concentration. Experimental conditions: MG-, MM- and GG-blocks concentration = 50 mg/L, Ca^2+^ concentration = 1 mM. TEP were stained with alcian blue.

TEP developed from MG-, MM- and GG-blocks at various Na^+^ concentrations with a fixed Ca^2+^ concentration of 1 mM were directly visualized by means of a light-field microscope. As can be seen in Fig. 4, at the Na^+^ concentration of 10 mM, the largest TEP in size and the highest concentration (Fig. 3) were found in the situation where GG-blocks served as precursor, with markedly complex and compact structure indicated by the strong blue color (Fig. 4c). On the contrary, TEP derived from MG- and MM-blocks were much smaller in size and less in concentration (Fig. 4a–b) than that derived from GG-blocks. Similar to the trends observed in Fig. 3, the microscopic images provide visual evidence that less TEP was generated with increasing Na^+^ concentration for all the three precursors studied due to the competition between sodium and calcium ions for the binding sites on the alginate blocks.

**Figure 5:**
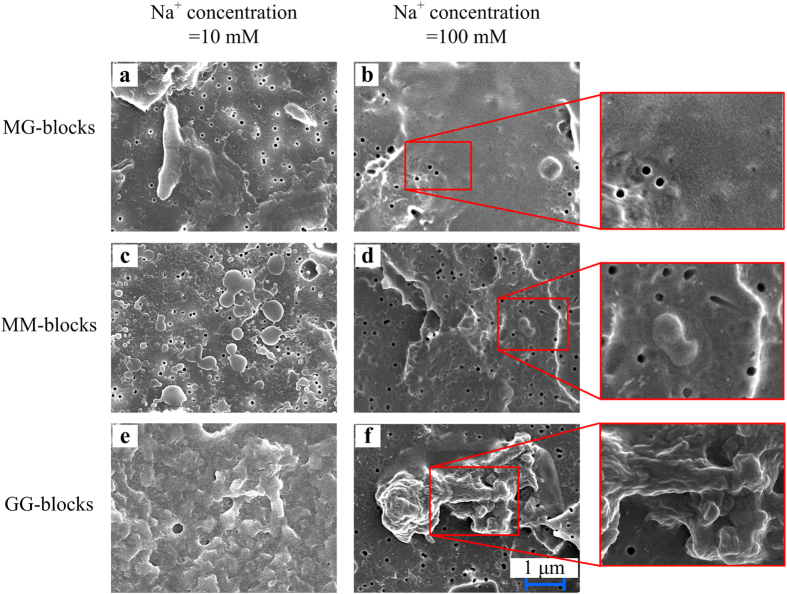
Micro-structure change of TEP derived from alginate blocks caused by increasing Na^+^ concentration. FESEM images of the micro-structure of TEP formed from MG-, MM- and GG-blocks at 10 mM (**a**,**c**,**e**) and 100 mM (**b**,**d**,**f**) Na^+^ concentration. Experimental conditions: MG-, MM- and GG-blocks concentration = 50 mg/L, Ca^2+^ concentration = 1 mM.

FESEM images (Fig. 5) further reveal the micro-structures of the TEP developed at various Na^+^ concentrations, which provide insights into the crosslink of precursors via calcium bonding. For example, at the Na^+^ concentration of 10 mM, some aggregated structure was observed for all the precursors studied, which probably resulted from the bridging or cross-linking effect of calcium ion among precursors. However, when the Na^+^ concentration was increased from 10 mM to 100 mM, nearly no TEP was developed from MG- and MM-blocks (Fig. 5b,d), whereas a significantly reduced amount of TEP-like aggregate was observed (Fig. 5f) in GG-blocks. Together with the results presented in Fig. 3, these microscopic observations offer strong and direct evidence in supporting the argument of that sodium ion at its elevated concentration can out-compete with calcium ion for the bonding sites in the precursors’ molecules, and such competition in turn suppresses the cross-linking among precursor molecules. In study of gelation of alginate solution, it had been reported that the formation of alginate gel beads was impossible at molar ratio of Na^+^ to Ca^2+^ of 97^30^, which is nearly the same ratio as used in this study at the Na^+^ concentration of 100 mM.

### Viscoelasticity of TEP developed at different chemistry conditions

**Figure 6:**
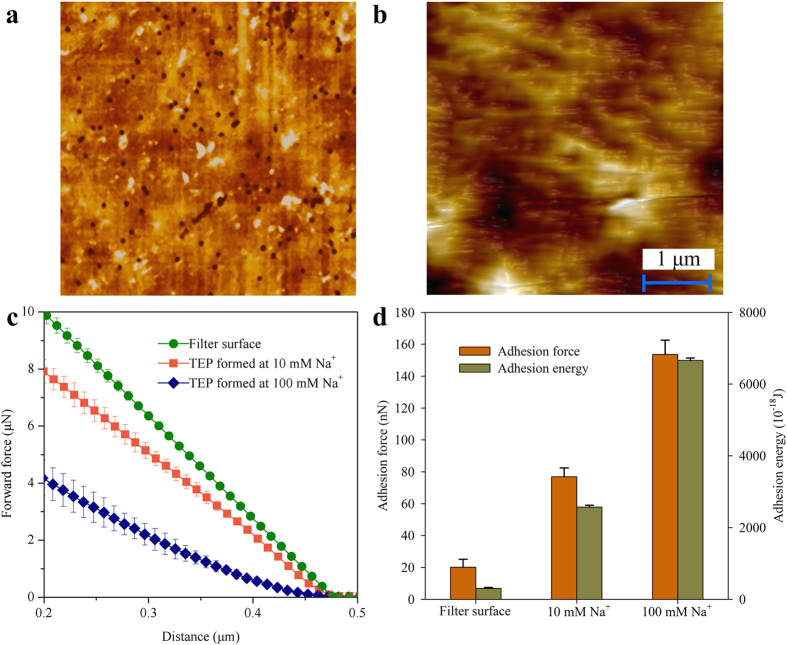
AFM forces measurements. AFM images of (**a**) clean filter surface and (**b**) TEP film retained on the filter. The (**c**) forward force curves and (**d**) maximum adhesion forces and total energies needed to completely pull off cantilever from the sample surfaces. Experimental conditions: GG-blocks concentration = 50 mg/L, Ca^2+^ concentration = 1 mM, TEP amount retained on the filters = 1 mg. Data are shown as the mean ± s.d., n = 50 independent measurements of the adhesion force and energy.

It has been known that TEP are highly sticky and deformable^5^,^9^,^11^. In this study, AFM was employed to measure adhesion force of the TEP layer, which provides quantitative information about the viscoelasticity and stickiness of TEP derived from precursors, i.e. GG-blocks at various Na^+^ concentrations with a fixed Ca^2+^ concentration. The force measurements were performed on the clean filter surface (Fig. 6a) and the filter surface covered by TEP (Fig. 6b), respectively. The elastic properties of a sample could be corroborated from the slope of the force curve. For a stiff surface, when the tip of cantilever touching the surface, the deflection of the tip is proportional to the distance between the tip and sample surface^31^,^32^ which is the case presented in Fig. 6a for the filter surface. However, as for soft surface, like the filter covered by TEP (Fig. 6b), the tip of the cantilever tends to indent the sample, resulting in a force curve with a smaller slope^31^,^32^. The forward force curves of the clean filter and TEP surfaces are shown in Fig. 6c. For force measurements on the clean filter surfaces, the 10 mM and 100 mM Na^+^ solutions were used and no notable difference lying in the force curves was found. For the TEP samples, it can be reasonably considered that the TEP derived from GG-blocks at the Na^+^ concentration of 100 mM should be much softer than the TEP generated from GG-blocks at the lower Na^+^ concentration of 10 mM. Increasing Na^+^ concentration at a fixed Ca^2+^ concentration led to a decreased stiffness of TEP. In addition, the adhesion force and energy were concurrently measured based on the retract force curves. As can be seen in Fig. 6d, higher adhesion force and energy are required to pull off the cantilever tip from the surface of TEP developed from GG-blocks as precursor at the higher Na^+^ concentration of 100 mM, indicating that the TEP formed at higher Na^+^ concentration appeared to be stickier than that formed at the lower Na^+^ concentration of 10 mM. These are likely due to the out-competing of Na^+^ against Ca^2+^, leading to lesser crosslinking degree among the precursor molecules. In the presence of calcium ion, a highly organized “egg-box” like structure of GG blocks can be developed^27^. However, increased Na^+^ concentration at a fixed Ca^2+^ concentration may out-compete with Ca^2+^ in forming this “egg-box” like structure of GG blocks. Therefore, less TEP was developed via the bonding between GG-blocks and Ca^2+^. Moreover, such TEP should possess a weaker crosslink and network structure that in turn explains increased softness and stickiness of TEP developed at high Na^+^ concentration as shown in Fig. 6. In fact, Ca-alginate beads developed at high Na^+^/Ca^2+^ ratio were found to be much softer than those formed at low Na^+^/Ca^2+^ condition^30^. Consequently, it appears that the quantity and quality of TEP produced from precursors studied are largely determined by the relative concentration of Na^+^ to Ca^2+^ present in aquatic environment.

### Mechanistic interpretation of TEP formation at various Na^+^/Ca^2+^ ratios

**Figure 7:**
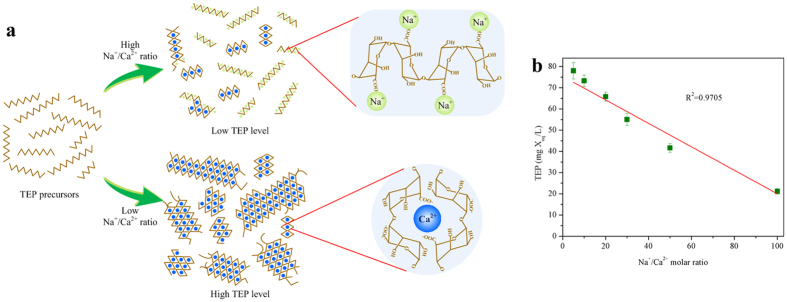
Mechanistic interpretation of TEP formation at various Na^+^/Ca^2+^ ratios. **(a)** Mechanistic interpretation of the structural changes occurring in GG-blocks bonding with calcium ions in competition with sodium ions. The blue and green dots represent the calcium ions and sodium ions respectively. The brown lines represent the GG-blocks. **(b)** The concentration of TEP derives from precursor materials as a function of Na^+^/Ca^2+^ molar ratio. Data are shown as the mean±s.d., n=6 independent measurements of the TEP concentrations.

As shown above, TEP formation from alginate blocks is significantly affected by calcium and sodium ions. Using GG-blocks as an example, Fig. 7 illustrates possible competition of Na^+^ against Ca^2+^ in bonding with precursor molecules. As discussed above, at the Na^+^ concentration of 10 mM, a fair amount of GG-blocks are cross-linked together through bridging by calcium ions, producing TEP with large size and complex structure. With the increase in the Na^+^ concentration from 10 mM to 100 mM, the bonding opportunity of GG-blocks to calcium ions is out-competed by sodium ions. As the result of such completion between Na^+^ and Ca^2+^, TEP formation was remarkably reduced as observed in Fig. 3. Moreover, a substantial amount of precursor molecules (e.g. GG-blocks) still remained as single molecules without inter-aggregation. This in turn may explain that TEP developed at the Na^+^ concentration of 100 mM were much smaller in size than those formed at 10 mM of Na^+^. It should also be pointed out that similar competition between Na^+^ and Ca^2+^ was also observed during the formation of alginate gel beads^33^,^34^. Chemically, similar mechanisms are applicable for TEP formation with MG- and MM-blocks as precursors.

## Conclusions

TEP formation was determined under various solution chemistries with alginate blocks as model precursors. The salient points derived from the present study are (i) TEP formation was dependent on physico-chemical properties of precursor materials at a given Na^+^/Ca^2+^ ratio, which determined the binding affinity and strength between precursors and calcium ions; (ii) Ca^2+^ was essential for the formation of TEP from precursor materials studied; (iii) Na^+^ itself had no contribution to the development of TEP, while high-concentration Na^+^ tended to out-compete Ca^2+^ for the binding sites on the precursor molecules, leading to a significantly decreased trend of TEP formation at high Na^+^/Ca^2+^ ratios and (iv) the AFM force measurements provided useful information on viscoelasticity of TEP formed at different Na^+^/Ca^2+^ ratios, revealing that the TEP formed at high Na^+^/Ca^2+^ ratio was softer and sticker than that formed at low Na^+^/Ca^2+^ ratio. The present study offers experimental evidence showing that a more abundant TEP can be expected in freshwater than in seawater. This in turn implies that TEP-associated biofilm development and membrane fouling would be more significant in membrane filtration of freshwater than seawater.

## Additional Information

**How to cite this article**: Meng, S. and Liu, Y. New insights into transparent exopolymer particles (TEP) formation from precursor materials at various Na_+_/Ca^2^^+^ ratios. *Sci. Rep.* **6**, 19747; doi: 10.1038/srep19747 (2016).
